# COVID-19: A Potential Cause of Non-convulsive Status Epilepticus

**DOI:** 10.7759/cureus.15041

**Published:** 2021-05-15

**Authors:** Karim El Aidaoui, Rita Ait Benhamou, Asmaa Hazim, Amal Haoudar, Chafik El Kettani

**Affiliations:** 1 Anesthesia and Critical Care, Cheikh Khalifa International University Hospital, Mohammed VI University of Health Sciences, Casablanca, MAR; 2 Neurology, Cheikh Khalifa International University Hospital, Mohammed VI University of Health Sciences, Casablanca, MAR

**Keywords:** covid-19, status epilepticus, seizure, confusion, sars-cov-2

## Abstract

Coronavirus disease 2019 (COVID-19) has been described as being primarily responsible for respiratory symptoms. Although several case reports have shown the importance of neurological manifestations, only a few have reported non-convulsive status epilepticus (NCSE) as the first manifestation of COVID-19 infection. Here, we report the case of a 30-year-old male patient with no past medical history who was admitted with altered consciousness. On examination, the patient had a Glasgow Coma Scale (GCS) of 13/15. Vital signs were within normal range. Computed tomography scan of the and magnetic resonance imaging of the brain were normal. Biochemical assessments showed a mild hyponatremia (134 mEq/L) and high levels of D-dimer and lactate dehydrogenase. Urine drug screening did not find any abnormality and a lumbar puncture showed an increased cerebrospinal fluid protein. The result of the reverse transcription polymerase chain reaction test in the nasopharyngeal swab was positive for severe acute respiratory syndrome coronavirus 2 (SARS-CoV-2). Electroencephalogram (EEG) showed a generalized epileptiform activity. Upon undergoing antiepileptic treatment, patient’s GCS improved to 15 gradually. A repeated EEG confirmed complete resolution of epileptic abnormalities four days later. This case report shows that SARS-CoV-2 infection can directly involve the central nervous system and can be manifested with isolated NCSE without any other neurological manifestations.

## Introduction

Neurological manifestations associated with coronavirus disease 2019 (COVID-19) are frequent and varied. While non-specific symptoms such as headache, dizziness, and myalgia have been described, more severe neurological disorders have been reported in other patients [1]. We report the case of a non-convulsive status epilepticus (NCSE) in a young patient as the only neurological manifestation of COVID-19 infection.

## Case presentation

A 30-year-old male patient with no past medical history was admitted on November 30th, 2020 with altered consciousness. Three days prior to his admission, he felt unwell and feverish. There was no documented seizure, cough, or dyspnea. On examination, the patient was drowsy and confused. His Glasgow Coma Scale (GCS) was 13/15 (E3V4M6). Pupils were equal and reactive to light. There was no focal neurological deficit. No cardiorespiratory distress or head injury were noticed. The patient’s vital signs were all within the normal range, including arterial pressure (132/66 mmHg), heart rate (82 beats per minute), and temperature (37.5°C).

The patient’s sodium level was low (134 mEq/L), which could not explain his neurological symptoms. The blood sugar level was within normal limits (156 mg/dL). Hematological and inflammatory assessment showed a high level of D-dimer (1,450 µg/L) and lactate dehydrogenase (317 U/L) with normal C-reactive protein (0.8 mg/L), procalcitonin (0.047 ng/mL), ferritin (166 µg/L), and troponin (0 ng/mL). Urine drug screening did not find any abnormality. Computed tomography (CT) scan of the head showed a non-specific left cerebellum hypodensity. Magnetic resonance imaging (MRI) of the brain which was done to complete the investigation did not reveal any abnormality.

In the current pandemic context, a fever episode made us think of an infection with severe acute respiratory syndrome coronavirus 2 (SARS-CoV-2). Therefore, we performed a reverse transcription polymerase chain reaction (RT-PCR) test in nasopharyngeal swab and a chest CT. The chest CT did not show lesions compatible with COVID-19 infection. Subsequently, the patient was admitted in the neurological intensive care unit. A lumbar puncture was performed. Cerebrospinal Fluid (CSF) analysis showed the following results: CSF glucose at 86 mg/dL, increased CSF protein at 54 mg/dL, CSF white blood cell count at 8/mm^3^ , CSF red blood cells count at 5/mm^3^, with no organisms in the culture. Serology for human immunodeficiency virus, viral hepatitis, and syphilis were all negative. India Ink preparation for *Cryptococcus *was negative in CSF. Twenty-four hours after admission, the RT-PCR result was positive for SARS-CoV-2.

**Figure 1:**
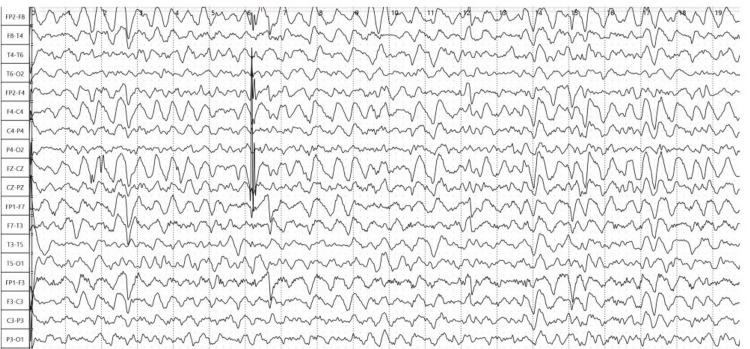
Electroencephalogram tracing showing a generalized status epilepticus.

During the patient’s hospital stay, hemodynamical and respiratory functions remained steady. However, his prolonged confusion remained unchanged. Therefore, a routine electroencephalogram (EEG) was performed, which showed a generalized epileptiform activity (Figure 1).

Upon undergoing antiepileptic treatment (midazolam 5 mg intravenous followed by levetiracetam 0.5 g twice daily), the patient gradually improved. He was no longer confused 24 hours later. A follow-up EEG confirmed complete resolution of epileptic abnormalities after four days. The patient was discharged home after one week, without any recurrence of neurological symptoms during the follow-up of three months.

## Discussion

In the beginning of the pandemic, COVID-19 was described as being primarily responsible for respiratory symptoms [2]. However, several studies have shown the existence of digestive, cardiac, and neurological manifestations [1,3]. Recently, neurological manifestations have been reported as an integral part of SARS-CoV-2 infection symptomatology [4]. Mao et al. [2] investigated 214 COVID-19 patients, one-third of whom had neurological manifestations at admission, including confusion, ataxia, seizures, and acute cerebrovascular disease. The detection of the virus in CSF using PCR technique has been described in several publications [5]. Other reports confirmed COVID-19 infection based on IgM and IgG antibodies in CSF [6]. It has been shown that SARS-CoV-2 binds to angiotensin-converting enzyme 2 (ACE2) membrane receptors present in the pulmonary and vascular endothelium as well as neuronal cells. One of the preferred entry ways for virus in the brain can be a retrograde neuronal dissemination via the olfactory mucosa, followed by the olfactory bulb [7]. Other authors have suggested that the destructive effects of COVID-19 in the central nervous system would be caused by the entry of pro-inflammatory cytokines from the periphery into the central nervous system, or the production of cytokines by astrocytes and activated microglia. Pro-inflammatory cytokines can cause blood-brain barrier disruption, increase glutamate levels, reduce gamma-aminobutyric acid (GABA) levels, and lead to epilepsy [8]. However, the exact way by which SARS-CoV-2 involves the central nervous and causes EEG abnormalities or seizures remains unclear [7,8].

In fact, an isolated alteration of vigilance with a progressive change in consciousness ranging from somnolence to coma evokes an encephalopathy. Even if multifactorial origin encephalopathy related to hypoxia or multiorgan damage has been described in COVID-19 among severely ill patients admitted to the intensive care unit [9], our patient did not develop respiratory symptoms of COVID-19. This goes against this theory. In addition, our patient had no previous history of seizures, and the mild hyponatremia cannot explain seizure occurrence. The clinical presentation of our patient suggests a NCSE presenting as confusional state. NCSE represents a prolonged state of seizures without obvious motor activity [10]. In our report, it is a case of absence status (AS), which is the most common form of NCSE. AS is based on clinical, electrophysiological (EEG), and therapeutic criteria [11]. In our view, the patient had a primary central nervous system disease related to COVID-19 that led to NCSE. More studies are needed to demonstrate the exact mechanism of seizures in COVID-19 patients.

## Conclusions

This case study reports a NCSE as the first manifestation of COVID-19 infection and shows that SARS-COV-2 can enter the central nervous system directly and cause NCSE in susceptible patients. Hence, COVID-19 infection can be manifested with isolated NCSE without any other neurological manifestations.
